# 4-(3,4-Dihydro-β-carbolin-1-yl)pyrimidin-2-amine

**DOI:** 10.1107/S160053680900600X

**Published:** 2009-02-25

**Authors:** Mat Ropi Mukhtar, Anissuhailin Zainal Abidin, Khalijah Awang, A. Hamid A. Hadi, Seik Weng Ng

**Affiliations:** a Department of Chemistry, University of Malaya, 50603 Kuala Lumpur, Malaysia

## Abstract

The mol­ecule of accanthomine A, C_15_H_13_N_5_, is approximately planar, with the indolyl fused-ring and the pyrimidyl ring being twisted by 31.7 (1)° The amino group of the five-membered ring is an intramolecular hydrogen-bond donor to a nitro­gen acceptor of the pyrimide ring. The amino group of the pyrimide ring is a hydrogen-bond donor to the N atoms of adjacent mol­ecules. These hydrogen-bonding inter­actions give rise to a layered network with a 4.8^2^ topology.

## Related literature

The β-carboline fragment is found in the crystal structures of two compounds that show selective CDK4-cycli D1 inhibitory activity; see: García *et al.* (2006 ▶). For related compounds, see: Costa *et al.* (2006 ▶); Kobayashi *et al.* (1995 ▶).
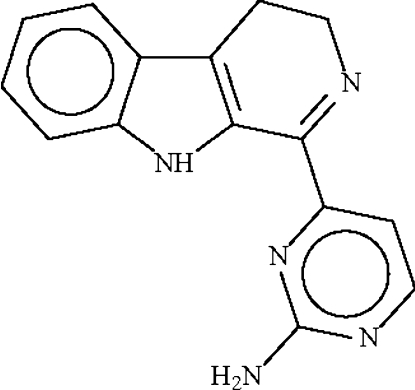


## Experimental

### 

#### Crystal data

C_15_H_13_N_5_
*M* *_r_* = 263.30
Monoclinic, 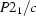

*a* = 11.4758 (2) Å
*b* = 12.6095 (2) Å
*c* = 8.9241 (2) Å
β = 102.116 (1)°
*V* = 1262.59 (4) Å^3^
*Z* = 4
Mo *K*α radiation
μ = 0.09 mm^−1^
*T* = 120 K
0.45 × 0.35 × 0.15 mm

#### Data collection

Bruker SMART APEX diffractometer
Absorption correction: none
11840 measured reflections
2905 independent reflections
2485 reflections with *I* > 2σ(*I*)
*R*_int_ = 0.026

#### Refinement

*R*[*F* ^2^ > 2σ(*F* ^2^)] = 0.037
*wR*(*F* ^2^) = 0.102
*S* = 1.02
2905 reflections
193 parameters
3 restraints
H atoms treated by a mixture of independent and constrained refinement
Δρ_max_ = 0.26 e Å^−3^
Δρ_min_ = −0.23 e Å^−3^

### 

Data collection: *APEX2* (Bruker, 2007 ▶); cell refinement: *SAINT* (Bruker, 2007 ▶); data reduction: *SAINT*; program(s) used to solve structure: *SHELXS97* (Sheldrick, 2008 ▶); program(s) used to refine structure: *SHELXL97* (Sheldrick, 2008 ▶); molecular graphics: *OLEX* (Dolomanov *et al.*, 2003 ▶) and *X-SEED* (Barbour, 2001 ▶); software used to prepare material for publication: *publCIF* (Westrip, 2009 ▶).

## Supplementary Material

Crystal structure: contains datablocks global, I. DOI: 10.1107/S160053680900600X/hg2480sup1.cif

Structure factors: contains datablocks I. DOI: 10.1107/S160053680900600X/hg2480Isup2.hkl

Additional supplementary materials:  crystallographic information; 3D view; checkCIF report

## Figures and Tables

**Table 1:** Hydrogen-bond geometry (Å, °)

*D*—H⋯*A*	*D*—H	H⋯*A*	*D*⋯*A*	*D*—H⋯*A*
N1—H11⋯N2^i^	0.89 (2)	2.14 (2)	2.994 (3)	161 (3)
N1—H12⋯N5^ii^	0.91 (3)	2.25 (3)	3.138 (3)	165 (3)
N4—H4⋯N3	0.88 (2)	2.29 (3)	2.825 (3)	119 (2)

Symmetry codes: (i) 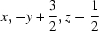; (ii) 
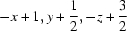
.

**Fig. 1.**
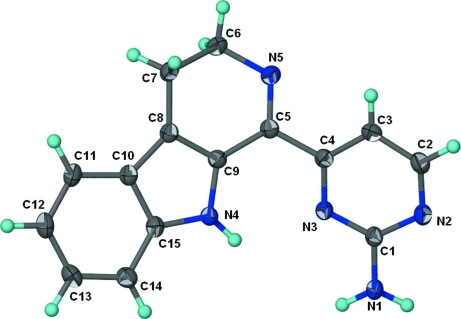
Thermal ellipsoid plot (Barbour, 2001) of C15H13N3 at the 70% probability level. Hydrogen atoms are drawn as spheres of arbitrary radius.

**Fig. 2.**
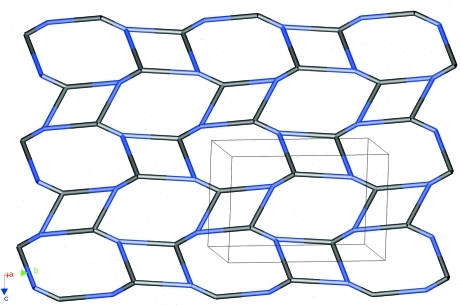
Layer structure (Dolomanov et al., 2003).

## supplementary crystallographic information

### Comment

The molecule of Accanthomine A (I) is approximately planar; the amino group of the five-membered ring is hydrogen-bond donor to a nitrogen acceptor of the pyrimidyl ring (Fig. 1). The amino group of the pyrimidyl ring is a hydrogen-bond donor to the nitrogen atoms of adjacent molecules. The hydrogen bonding interactions give rise to a layer network with a 4.8 (2) topology (Fig. 2).

### Experimental

*Litsea machilifolia* was collected from the Mukim Telang Reserve, Kuala Lipis, Pahang. Specimens (KL5459) were deposited at the herbarium, Department of Chemistry, University of Malaya.

Dried and grounded leaves of *Litsea machilifolia* (2.1 kg) were extracted with dichloromethane. The dichloromethane extract was concentrated under reduced pressure to a volume of 500 ml and this was repeatedly extracted with 5% hydrocloric acid. The combined extracts were then basified with 10% ammonium hydroxide to a pH 11, and then re-extracted with dichloromethane. The crude alkaloid fraction was dark brown (4.0 g). A portion (4.0 g) was subjected to column chromatography on silica gel 60 GF_254_ by using a step gradient of dichloromethane and methanol. One of the fractions when further purified by CC with 100% dichloromethane afforded the pure compound, accanthomine A (8 mg), whose formulation was established by NMR spectroscopic analysis.

### Refinement

Carbon-bound H-atoms were placed in calculated positions (C–H 0.95–0.99 Å) and were included in the refinement in the riding model approximation, with *U*(H) set to 1.2*U*(C). The nitrogen-bound H-atoms were located in a difference Fourier map, and were refined with a distance restraint of N–H 0.88±0.01 Å; their temperature factors were freely refined.

### Crystal data

**Table:**

C_15_H_13_N_5_	*F*(000) = 552
*M**_r_* = 263.30	*D*_x_ = 1.385 Mg m^−^^3^
Monoclinic, *P*2_1_/*c*	Mo *K*α radiation, λ = 0.71073 Å
Hall symbol: -P 2ybc	Cell parameters from 4559 reflections
*a* = 11.4758 (2) Å	θ = 2.4–28.3°
*b* = 12.6095 (2) Å	µ = 0.09 mm^−^^1^
*c* = 8.9241 (2) Å	*T* = 120 K
β = 102.116 (1)°	Irregular block, light brown
*V* = 1262.59 (4) Å^3^	0.45 × 0.35 × 0.15 mm
*Z* = 4	

### Data collection

**Table:**

Bruker SMART APEX diffractometer	2485 reflections with *I* > 2σ(*I*)
Radiation source: fine-focus sealed tube	*R*_int_ = 0.026
graphite	θ_max_ = 27.5°, θ_min_ = 1.8°
ω scans	*h* = −14→14
11840 measured reflections	*k* = −16→16
2905 independent reflections	*l* = −11→11

### Refinement

**Table:**

Refinement on *F*^2^	Primary atom site location: structure-invariant direct methods
Least-squares matrix: full	Secondary atom site location: difference Fourier map
*R*[*F*^2^ > 2σ(*F*^2^)] = 0.037	Hydrogen site location: inferred from neighbouring sites
*wR*(*F*^2^) = 0.102	H atoms treated by a mixture of independent and constrained refinement
*S* = 1.02	*w* = 1/[σ^2^(*F*_o_^2^) + (0.0591*P*)^2^ + 0.3611*P*] where *P* = (*F*_o_^2^ + 2*F*_c_^2^)/3
2905 reflections	(Δ/σ)_max_ = 0.001
193 parameters	Δρ_max_ = 0.26 e Å^−^^3^
3 restraints	Δρ_min_ = −0.23 e Å^−^^3^

### Fractional atomic coordinates and isotropic or equivalent isotropic displacement parameters (Å^2^)

**Table:**

	*x*	*y*	*z*	*U*_iso_*/*U*_eq_	
N1	0.50153 (18)	0.76301 (16)	0.7128 (2)	0.0209 (4)	
N2	0.55480 (17)	0.65191 (15)	0.9213 (2)	0.0187 (4)	
N3	0.60199 (16)	0.61005 (14)	0.6777 (2)	0.0165 (4)	
N4	0.73723 (17)	0.59739 (15)	0.4478 (2)	0.0180 (4)	
C1	0.55292 (19)	0.67210 (17)	0.7718 (2)	0.0163 (4)	
C2	0.6123 (2)	0.56393 (18)	0.9781 (3)	0.0194 (5)	
H2	0.6172	0.5480	1.0833	0.023*	
C3	0.6649 (2)	0.49480 (17)	0.8924 (3)	0.0187 (5)	
H3	0.7049	0.4325	0.9356	0.022*	
C4	0.65609 (18)	0.52161 (17)	0.7390 (2)	0.0159 (4)	
C5	0.70796 (18)	0.45156 (17)	0.6351 (2)	0.0162 (4)	
C6	0.7651 (2)	0.28242 (17)	0.5636 (3)	0.0199 (5)	
H6A	0.6977	0.2605	0.4809	0.024*	
H6B	0.7970	0.2176	0.6203	0.024*	
C7	0.8626 (2)	0.32896 (17)	0.4896 (3)	0.0185 (5)	
H7A	0.9393	0.3309	0.5650	0.022*	
H7B	0.8728	0.2843	0.4020	0.022*	
C8	0.82678 (19)	0.43888 (17)	0.4355 (2)	0.0165 (4)	
C9	0.75008 (19)	0.49495 (17)	0.5047 (2)	0.0162 (4)	
C10	0.86494 (19)	0.50915 (17)	0.3305 (2)	0.0168 (5)	
C11	0.9446 (2)	0.50053 (18)	0.2302 (3)	0.0203 (5)	
H11A	0.9863	0.4362	0.2233	0.024*	
C12	0.9608 (2)	0.58717 (19)	0.1426 (3)	0.0227 (5)	
H12A	1.0146	0.5823	0.0751	0.027*	
C13	0.8991 (2)	0.68278 (19)	0.1512 (3)	0.0218 (5)	
H13	0.9111	0.7406	0.0879	0.026*	
C14	0.8217 (2)	0.69440 (18)	0.2493 (3)	0.0202 (5)	
H14	0.7809	0.7593	0.2554	0.024*	
C15	0.80577 (19)	0.60715 (17)	0.3395 (2)	0.0173 (5)	
N5	0.71831 (17)	0.35226 (14)	0.6694 (2)	0.0188 (4)	
H11	0.503 (3)	0.778 (2)	0.616 (2)	0.030 (8)*	
H12	0.448 (3)	0.796 (2)	0.759 (4)	0.031 (8)*	
H4	0.686 (2)	0.643 (2)	0.472 (3)	0.031 (8)*	

### Atomic displacement parameters (Å^2^)

**Table:**

	*U*^11^	*U*^22^	*U*^33^	*U*^12^	*U*^13^	*U*^23^
N1	0.0250 (10)	0.0207 (10)	0.0199 (10)	0.0063 (8)	0.0115 (8)	0.0024 (7)
N2	0.0208 (9)	0.0199 (9)	0.0167 (9)	−0.0008 (7)	0.0067 (7)	−0.0013 (7)
N3	0.0172 (9)	0.0161 (9)	0.0179 (9)	−0.0004 (7)	0.0072 (7)	−0.0004 (7)
N4	0.0193 (9)	0.0169 (9)	0.0202 (9)	0.0031 (7)	0.0097 (7)	0.0028 (7)
C1	0.0152 (10)	0.0174 (10)	0.0175 (10)	−0.0027 (8)	0.0061 (8)	−0.0010 (8)
C2	0.0218 (11)	0.0208 (11)	0.0157 (10)	−0.0030 (9)	0.0043 (8)	0.0004 (8)
C3	0.0214 (11)	0.0159 (10)	0.0192 (11)	−0.0006 (8)	0.0050 (9)	0.0020 (8)
C4	0.0142 (9)	0.0153 (10)	0.0191 (11)	−0.0032 (8)	0.0057 (8)	−0.0005 (8)
C5	0.0145 (9)	0.0166 (10)	0.0178 (10)	−0.0009 (8)	0.0040 (8)	0.0002 (8)
C6	0.0237 (11)	0.0151 (10)	0.0217 (11)	−0.0001 (8)	0.0065 (9)	−0.0018 (8)
C7	0.0197 (10)	0.0176 (10)	0.0187 (10)	0.0019 (8)	0.0055 (8)	−0.0023 (8)
C8	0.0163 (10)	0.0171 (10)	0.0160 (10)	−0.0005 (8)	0.0036 (8)	−0.0015 (8)
C9	0.0162 (10)	0.0155 (10)	0.0175 (10)	−0.0006 (8)	0.0045 (8)	0.0001 (8)
C10	0.0164 (10)	0.0188 (10)	0.0154 (10)	−0.0006 (8)	0.0035 (8)	−0.0012 (8)
C11	0.0203 (11)	0.0226 (11)	0.0195 (11)	0.0000 (9)	0.0077 (9)	−0.0041 (8)
C12	0.0219 (11)	0.0289 (12)	0.0196 (11)	−0.0024 (9)	0.0095 (9)	−0.0021 (9)
C13	0.0213 (11)	0.0261 (12)	0.0186 (11)	−0.0029 (9)	0.0054 (9)	0.0040 (9)
C14	0.0187 (10)	0.0210 (11)	0.0214 (11)	0.0019 (9)	0.0053 (9)	0.0032 (9)
C15	0.0154 (10)	0.0205 (11)	0.0165 (10)	−0.0002 (8)	0.0046 (8)	−0.0006 (8)
N5	0.0204 (9)	0.0160 (9)	0.0209 (9)	−0.0001 (7)	0.0068 (7)	−0.0009 (7)

### Geometric parameters (Å, °)

**Table:**

N1—C1	1.345 (3)	C6—C7	1.531 (3)
N1—H11	0.894 (17)	C6—H6A	0.9900
N1—H12	0.91 (3)	C6—H6B	0.9900
N2—C2	1.335 (3)	C7—C8	1.497 (3)
N2—C1	1.354 (3)	C7—H7A	0.9900
N3—C4	1.336 (3)	C7—H7B	0.9900
N3—C1	1.354 (3)	C8—C9	1.373 (3)
N4—C15	1.374 (3)	C8—C10	1.424 (3)
N4—C9	1.384 (3)	C10—C11	1.411 (3)
N4—H4	0.883 (17)	C10—C15	1.420 (3)
C2—C3	1.380 (3)	C11—C12	1.379 (3)
C2—H2	0.9500	C11—H11A	0.9500
C3—C4	1.393 (3)	C12—C13	1.408 (3)
C3—H3	0.9500	C12—H12A	0.9500
C4—C5	1.492 (3)	C13—C14	1.380 (3)
C5—N5	1.288 (3)	C13—H13	0.9500
C5—C9	1.457 (3)	C14—C15	1.398 (3)
C6—N5	1.472 (3)	C14—H14	0.9500
			
C1—N1—H11	117.6 (19)	C8—C7—H7A	110.0
C1—N1—H12	120.0 (19)	C6—C7—H7A	110.0
H11—N1—H12	120 (3)	C8—C7—H7B	110.0
C2—N2—C1	115.78 (18)	C6—C7—H7B	110.0
C4—N3—C1	116.49 (18)	H7A—C7—H7B	108.4
C15—N4—C9	108.00 (17)	C9—C8—C10	106.88 (19)
C15—N4—H4	128 (2)	C9—C8—C7	119.37 (19)
C9—N4—H4	123 (2)	C10—C8—C7	133.41 (19)
N1—C1—N2	117.46 (19)	C8—C9—N4	110.16 (18)
N1—C1—N3	117.05 (19)	C8—C9—C5	121.2 (2)
N2—C1—N3	125.5 (2)	N4—C9—C5	127.99 (19)
N2—C2—C3	123.6 (2)	C11—C10—C15	119.0 (2)
N2—C2—H2	118.2	C11—C10—C8	134.3 (2)
C3—C2—H2	118.2	C15—C10—C8	106.69 (18)
C2—C3—C4	116.2 (2)	C12—C11—C10	118.7 (2)
C2—C3—H3	121.9	C12—C11—H11A	120.6
C4—C3—H3	121.9	C10—C11—H11A	120.6
N3—C4—C3	122.46 (19)	C11—C12—C13	121.2 (2)
N3—C4—C5	116.83 (18)	C11—C12—H12A	119.4
C3—C4—C5	120.71 (19)	C13—C12—H12A	119.4
N5—C5—C9	121.66 (19)	C14—C13—C12	121.6 (2)
N5—C5—C4	117.19 (19)	C14—C13—H13	119.2
C9—C5—C4	121.09 (19)	C12—C13—H13	119.2
N5—C6—C7	116.45 (18)	C13—C14—C15	117.5 (2)
N5—C6—H6A	108.2	C13—C14—H14	121.3
C7—C6—H6A	108.2	C15—C14—H14	121.3
N5—C6—H6B	108.2	N4—C15—C14	129.7 (2)
C7—C6—H6B	108.2	N4—C15—C10	108.25 (18)
H6A—C6—H6B	107.3	C14—C15—C10	122.0 (2)
C8—C7—C6	108.57 (17)	C5—N5—C6	117.20 (18)
			
C2—N2—C1—N1	−176.44 (19)	C4—C5—C9—C8	161.4 (2)
C2—N2—C1—N3	1.3 (3)	N5—C5—C9—N4	174.8 (2)
C4—N3—C1—N1	177.94 (19)	C4—C5—C9—N4	−8.1 (3)
C4—N3—C1—N2	0.2 (3)	C9—C8—C10—C11	177.9 (2)
C1—N2—C2—C3	−1.5 (3)	C7—C8—C10—C11	4.9 (4)
N2—C2—C3—C4	0.4 (3)	C9—C8—C10—C15	−1.1 (2)
C1—N3—C4—C3	−1.5 (3)	C7—C8—C10—C15	−174.0 (2)
C1—N3—C4—C5	178.65 (18)	C15—C10—C11—C12	−1.1 (3)
C2—C3—C4—N3	1.3 (3)	C8—C10—C11—C12	−179.9 (2)
C2—C3—C4—C5	−178.92 (19)	C10—C11—C12—C13	−0.3 (3)
N3—C4—C5—N5	−154.0 (2)	C11—C12—C13—C14	1.2 (4)
C3—C4—C5—N5	26.2 (3)	C12—C13—C14—C15	−0.6 (3)
N3—C4—C5—C9	28.7 (3)	C9—N4—C15—C14	179.5 (2)
C3—C4—C5—C9	−151.1 (2)	C9—N4—C15—C10	−1.2 (2)
N5—C6—C7—C8	−44.9 (3)	C13—C14—C15—N4	178.4 (2)
C6—C7—C8—C9	25.0 (3)	C13—C14—C15—C10	−0.9 (3)
C6—C7—C8—C10	−162.7 (2)	C11—C10—C15—N4	−177.72 (19)
C10—C8—C9—N4	0.4 (3)	C8—C10—C15—N4	1.4 (2)
C7—C8—C9—N4	174.49 (18)	C11—C10—C15—C14	1.7 (3)
C10—C8—C9—C5	−170.84 (19)	C8—C10—C15—C14	−179.2 (2)
C7—C8—C9—C5	3.3 (3)	C9—C5—N5—C6	−5.1 (3)
C15—N4—C9—C8	0.5 (3)	C4—C5—N5—C6	177.67 (18)
C15—N4—C9—C5	171.0 (2)	C7—C6—N5—C5	36.6 (3)
N5—C5—C9—C8	−15.7 (3)		

### Hydrogen-bond geometry (Å, °)

**Table:**

*D*—H···*A*	*D*—H	H···*A*	*D*···*A*	*D*—H···*A*
N1—H11···N2^i^	0.89 (2)	2.14 (2)	2.994 (3)	161 (3)
N1—H12···N5^ii^	0.91 (3)	2.25 (3)	3.138 (3)	165 (3)
N4—H4···N3	0.88 (2)	2.29 (3)	2.825 (3)	119 (2)

Symmetry codes: (i) *x*, −*y*+3/2, *z*−1/2; (ii) −*x*+1, *y*+1/2, −*z*+3/2.
